# Concurrent material and structure optimization of multiphase hierarchical systems within a continuum micromechanics framework

**DOI:** 10.1007/s00158-021-02907-1

**Published:** 2021-05-31

**Authors:** Tarun Gangwar, Dominik Schillinger

**Affiliations:** 1 Department of Civil, Environmental, and Geo-Engineering, University of Minnesota, Twin Cities, USA; 2 Institute of Mechanics and Computational Mechanics, Leibniz Universität Hannover, Hannover, Germany

**Keywords:** Multiphase topology optimization, Concurrent design, Continuum micromechanics, Homogenization, Hierarchical systems, Sensitivity analysis

## Abstract

We present a concurrent material and structure optimization framework for multiphase hierarchical systems that relies on homogenization estimates based on continuum micromechanics to account for material behavior across many different length scales. We show that the analytical nature of these estimates enables material optimization via a series of inexpensive “discretization-free” constraint optimization problems whose computational cost is independent of the number of hierarchical scales involved. To illustrate the strength of this unique property, we define new benchmark tests with several material scales that for the first time become computationally feasible via our framework. We also outline its potential in engineering applications by reproducing self-optimizing mechanisms in the natural hierarchical system of bamboo culm tissue.

## Introduction

Natural materials such as wood, bone, or rocks and soils (Wegst et al. 2015; Zheng et al. 2014) can be considered multiphase and multiscale systems, whose multiphase composition evolves over multiple length scales, with heterogeneities ranging from micrometers to centimeters. Their complex multiphase hierarchical organization in conjunction with mechanical, physiological and reproductive constraints poses significant challenges for the study of their behavior. In particular, natural materials develop self-optimizing mechanisms across multiple scales, driven by the environment in which they are created (Wölf 1986; Gibson 2012; Gao et al. 2003). A rational understanding of these mechanisms help pave the way forward to many engineering applications such as the genetic tailoring of crops (Brulé et al. 2016; McCann et al. 2014), bone remodeling and patient-specific diagnostic simulations (Rodrigues et al. 2002b; Blanchard et al. 2016; Nguyen et al. 2017), and the fabrication of bioinspired engineering materials (Wegst et al. 2015; Holstov et al. 2015).

In the literature, one can find several classes of methods that work towards that goal. Substantial progress has been made over the past three decades in topology optimization methods (Bendsøe and Kikuchi 1988; Bendsøe 1989; Bendsøe and Sigmund 2013; Wang et al. 2003; Sigmund and Maute 2013), which have been extended to optimize multiscale systems (Coelho et al. 2009; Radman et al. 2013; Cadman et al. 2013; Gao et al. 2019; Wang and Wang 2004). In this context, integrating homogenization in topology optimization is a well-established concept, often applied in conjunction with relaxation to ill-defined 0-1 type problems (Bendsøe and Sigmund 2013; 1999; Hassani and Hinton 1998; Allaire and Aubry 1999) and implemented through computational homogenization or “unit-cell” methods (Fish 2013; Michel et al. 1999; Guedes and Kikuchi 1990; Fritzen et al. 2016; Xia and Breitkopf 2017). The increase in design variables, however, driven exponentially with each additional scale, restricts existing methods to simple scenarios with essentially no more than two scales.

The idea of concurrent multiscale analysis and topology optimization (Xia and Breitkopf 2014; 2015; Rodrigues et al. 2002a; Coelho et al. 2008; Nakshatrala et al. 2013) is to divide the multiscale problem into two nested sub-problems, one at the macroscale (structure) and the other at the microscale (material). At each macroscale material point, the microstructure is optimized under macroscale influence. In turn, the microscale sub-problems at material points provide the constitutive material behavior for the macroscale structure optimization problem. Due to their large computational cost, existing methods are limited to small two-scale problems. In particular, they are unable to handle multiphase hierarchical systems. We note that as a first step out of this dilemma, the material sub-problem has been formulated in the context of rule-of-mixture-based homogenization methods (Jog et al. 1994; Theocaris and Stavroulakis 1999).

From a multiscale analysis viewpoint, the cost for resolving hierarchical scales computationally, e.g., through multiscale finite elements (Efendiev et al. 2013; Nguyen and Schillinger 2019a; 2019b) or computational homogenization (Yuan and Fish 2009; Le et al. 2015; Liu et al. 2016; Bessa et al. 2017), increases exponentially with each additional scale, making the computational treatment of multiphase hierarchical systems prohibitively expensive. Continuum micromechanics provides a rigorous framework to analytically transfer statistical information of multiphase hierarchical systems, such as volume fraction, shape of constituents, and interaction between constituents into *estimates* of associated macroscale properties (Zaoui 2002; Suquet 2014). Continuum micromechanics-based homogenization has been successfully applied to describe natural multiscale systems such as wood, bone, or cement (Fritsch and Hellmich 2007; Fritsch et al. 2009; Pichler and Hellmich 2011; Morin et al. 2017; Hofstetter et al. 2005). In our recent work (Gangwar and Schillinger 2019; Gangwar et al. 2020), we showed that continuum micromechanics models can accurately predict both linear elastic and inelastic behavior of plant materials. We also demonstrated that each hierarchical level can be statistically characterized through microimaging techniques.

In this article, we combine well-established and mature results from the continuum micromechanics and topology optimization frontiers to derive an efficient concurrent material and structure optimization method that can tackle the computing challenge of optimizing multiphase hierarchical systems. Our method is based on the division of the compliance minimization problem in two sub-problems, utilizing the pointwise definition of material design variables. The master problem optimizes the macroscale distribution of a set of materials, whereas slave problems at each material point optimize homogenized properties with respect to microscale design variables expressed within a continuum mechanics framework.

Our article is structured as follows. In Section 2, we briefly review relevant principles of continuum micromechanics in the light of multiscale topology optimization. In Section 3, we discuss the concurrent material and structure optimization formulation, including a definition of the admissible design space for both sub-problems. In Section 4, we discuss the finite element discretization of the master problem and the implementation of both master and slave problems within a general optimization algorithm. In Section 5, we define new test problems that illustrate the efficiency of our method, and apply our framework for understanding self-optimizing mechanisms of bamboo culm. We close with a summary and outlook in Section 6.

## Multiscaling concepts in continuum micromechanics

Continuum micromechanics forms a rigorous foundation for the analytical estimation of homogenized properties of hierarchical systems with random microstructures. In this section, we briefly review basic multiscale analysis principles that we will use later in the context of concurrent material and structure optimization.

### Foundation principles

**Fig. 1:**
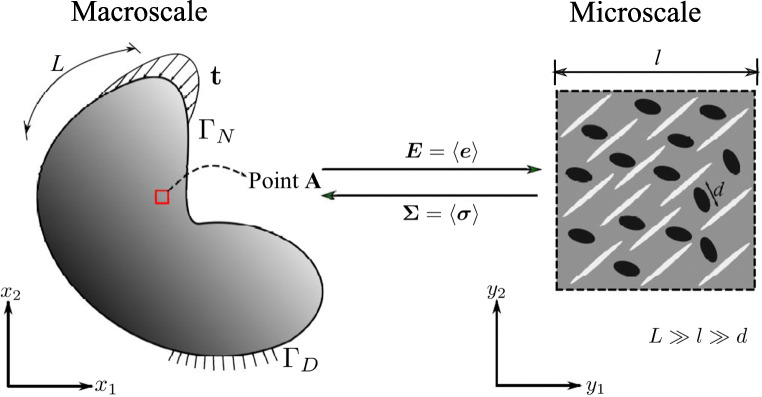
Homogenization and multiscale principles

The goal of any homogenization method is to replace the actual complex heterogeneous medium with a fictitious homogeneous one that has equivalent global behavior (Zaoui 2002; Suquet 2014). Figure 1 illustrates the key concepts. An important objective is to establish an “equivalent homogeneous element” whose mechanical response is equivalent to a representative volume element (RVE) of the microheterogeneous material. For the existence of such an RVE, a minimal requirement is that the characteristic length, *d*, of the considered inhomogeneities and deformation mechanisms is much smaller than the size, *l*, of the RVE. As a consequence, the RVE can be considered representative of the material in the macroscaleally homogeneous body (see Fig. 1). Moreover, *l* must be much smaller than the characteristic length scale of the variation in the loading on the macroscale structure, *L*. Therefore, proper scale separation implies that:

1 $$ d \ll l \ll L.  $$

We start with the variational form of the macroscale boundary value problem defined on a domain *Ω* as shown in Fig. 1. The domain is subjected to traction ***t*** at the Neumann boundary *Γ*_*N*_ and prescribed displacements at the Dirichlet boundary *Γ*_*D*_ with a body force field ***b***. The weak form states: Find a macroscale displacement field $ \boldsymbol {\bar {u}} \in U $ such that

2 $$ {\int}_{{\varOmega}} \boldsymbol{{\varSigma}}(\boldsymbol{\bar{u}}):\boldsymbol{E}(\boldsymbol{v})  d{\varOmega} = {\int}_{{\varOmega}} \boldsymbol{b} \cdot \boldsymbol{v} d{\varOmega} + {\int}_{{{\varGamma}}_{N}} \boldsymbol{t} \cdot \boldsymbol{v} ds,   \forall \boldsymbol{v} \in U,  $$

where the space *U* of test and trial functions is kinematically admissible. A constitutive relation between the stress **Σ** and the strain ***E*** will close this boundary value problem.

Figure 1 schematically illustrates the homogenization framework for establishing the relation between **Σ** and ***E***. The macroscale strain tensor ***E*** is calculated for each material point in the domain *Ω*. Next, ***E*** is utilized to formulate boundary conditions imposed on the microscale RVE. A numerical solution or an analytical estimate of the microscale boundary value problem will provide the macroscale stress tensor **Σ**. The nature of boundary conditions on the microscale RVE is unknown, and that makes the microscale boundary value problem an “ill-posed” problem. Assumptions on the boundary conditions have to be made to define this boundary value problem.

### Microscale problem and choice of boundary conditions

According to the *homogeneous strain boundary conditions*, the RVE is subjected to prescribed surface displacements $\boldsymbol {u}^{g}(\boldsymbol {x},\bar {\boldsymbol {y}})$ at the boundary such that:

3 $$ \boldsymbol{u}^{g}(\boldsymbol{x},\bar{\boldsymbol{y}}) = \boldsymbol{E}(\boldsymbol{x}) \cdot \bar{\boldsymbol{y}}.  $$

Here, any field *f*(***x***, ***y***) denotes a microstructural field variation in the RVE domain *Ω*_*y*_ situated at a macroscale material point ***x***. The position vector at the boundary of the RVE is denoted by $ \bar {\boldsymbol {y}} $. The corresponding kinematically compatible microscale trial strain field ***e***(***x***, ***y***) inside the RVE fulfills an equivalent volume average as:

4 $$ \langle \boldsymbol{e}(\boldsymbol{x},\boldsymbol{y}) \rangle_{{\varOmega}_{y}} = \frac{1}{|{\varOmega}_{y} |}{\int}_{{\varOmega}_{y}} \boldsymbol{e}(\boldsymbol{x},\boldsymbol{y}) d{\varOmega}_{y} = \boldsymbol{E} (\boldsymbol{x}).  $$

Similarly, *homogeneous stress boundary conditions* rely on surface tractions $ T^{g} (\boldsymbol {x},\bar {\boldsymbol {y}}) $ that are prescribed at the boundary and produce a constant stress **Σ**(***x***) in the fictitious homogeneous material at a point ***x***:

5 $$ T^{g}(\boldsymbol{x},\bar{\boldsymbol{y}}) = \boldsymbol{{\varSigma}} (\boldsymbol{x}) \cdot n ,  $$

where *n* is the unit outward normal at the boundary of the RVE. Any equilibrated trial stress field ***τ***(***x***, ***y***) in the RVE, that is, ∇_*y*_ ⋅***τ***(***x***, ***y***) = 0, obeys:

6 $$ \langle \boldsymbol{\tau}(\boldsymbol{x},\boldsymbol{y}) \rangle_{{\varOmega}_{y}} = \frac{1}{| {\varOmega}_{y} |}{\int}_{{\varOmega}_{y}} \boldsymbol{\tau}(\boldsymbol{x},\boldsymbol{y}) d{\varOmega}_{y} = \boldsymbol{{\varSigma}} (\boldsymbol{x}).  $$

We assume that all constituent phases in the RVE are linear elastic and perfectly bonded with each other. This assumption allows us to define a strain energy potential  inside the RVE domain *Ω*_*y*_ as:
7where  defines the linear elastic tensor at the microscale RVE situated at the macroscale material point ***x***. The principle of minimum potential energy at the microscale RVE is based on the actual strain field *ε* in the RVE as:
8where  is the set of kinematically admissible trial strain fields following the homogeneous strain boundary conditions (3) and (4).

For the linear elastic constituent phases, the effective strain energy potential  at the macroscale is:
9where  is the homogenized stiffness tensor at the macroscale material point ***x***. Following (8), (9), and Hill’s Lemma, we conclude:
10Relation (10) bridges macro- and microscales. Given a complete material and geometric description of the RVE, (10) can be solved numerically. In the case of partial statistical information, however, only suitable estimates to  can be obtained, which we summarize in the following subsection. We can also derive an equivalent statement to (10) for the complementary stress potential with the statically admissible trial stress field set  as:
11

### Homogenization based on Eshelby’s analytical solution

The linear constitutive relations for the constituent phases in the RVE imply that the trial strain and stress fields (***e***, ***τ***) must be linear and homogeneous with respect to ***E*** and **Σ**. Therefore, ***e*** and ***τ*** can be written in terms of the strain and stress concentration tensors  and  as:
12Using these relations in (10) and (11), we arrive at the following bounds:
13a13bIt is clear from (13a) that the estimation of the concentration tensors  and  will result in the upper and lower bound for the homogenized stiffness . The simplest choice for  and  is to assume a uniform strain or stress state throughout the RVE, i.e.,  or , where  is a fourth-order symmetric unit tensor. This choice leads to the well-known Voigt and Reuss estimates, which have been used in topology optimization as an interpolation between solid and void (Swan and Kosaka 1997a, 1997b). However, the Voigt-Reuss bounds do not consider any other statistical information beyond the volume fraction.

Homogenization schemes based on Eshelby’s matrix-inclusion solutions can incorporate the volume fraction, the shape of phases, and their interaction with each other. Eshelby’s problem relates strains in an ellipsoidal inclusion perfectly bonded with the surrounded homogeneous infinite elastic matrix to the applied homogeneous strains at infinity. We denote the elastic moduli of the ellipsoidal inclusion and the matrix as  and , respectively. The strains in the inclusion in response to the homogeneous strain ***E***^0^ at infinity are uniform. The uniform strain field *ε*_*H*_ in the inclusion is:
14The Hill tensor  characterizes the morphology of the inclusion and its interaction with the surrounding matrix.  depends on the shape and orientation of the inclusion as well as the stiffness tensor of the reference matrix . Analytical expressions for  can be found in Laws (1977), Laws (1985), and Masson (2008).

An important consequence of Eshelby’s analytical solution is that the strain field in the inclusion is uniform. Given the uniform stiffness moduli of the phases in the RVE, we can replace the stress and strain fields in the phases with the average stress and strain values ***σ***_*r*_ and *ε*_*r*_. Following (4) and (6), we write ***E*** and **Σ** in terms of *ε*_*r*_ and ***σ***_*r*_ as:

15 $$ \boldsymbol{E} = \sum\limits_{r} \phi_{r} {\varepsilon}_{r}   \text{and}   \boldsymbol{{\varSigma}} = \sum\limits_{r} \phi_{r} \boldsymbol{\sigma}_{r},  $$

where *ϕ*_*r*_ is the volume fraction of the phase *r*. Following (12), we can relate the average micro-strain *ε*_*r*_ and the macro-strain ***E*** via an average concentration strain tensor :
16We combine (15) and (16) with the phase constitutive relation . Comparison with the macroscale constitutive relation  yields the homogenized estimate of stiffness in terms of the volume fraction, stiffness, and localization tensor of constituent phases as:
17

For the estimation of , we approximate the average strains in each phase *r* by the inclusion strains *ε*_*H*_ in (14), i.e., *ε*_*r*_ = *ε*_*H*_. It implies that the average strains *ε*_*r*_ in each phase of the RVE are considered equal to those of an ellipsoidal inhomogeneity with the phase stiffness , embedded in a fictitious infinite matrix with stiffness , subjected to some homogeneous strain ***E***^0^ applied at infinity. Using the strain average rule in (15), we find a relation between the homogenized macro-strain ***E*** and the homogeneous strain ***E***^0^ at infinity in the fictitious matrix as:
18

With *ε*_*r*_ = *ε*_*H*_, the substitution of ***E***^0^ in (14) and the comparison with (16) yields the following estimate of the concentration strain tensor :
19The homogenized stiffness  follows from (17) as:
20where *s* is a free index for each phase in the RVE. Expression (20) relies on the statistical characterization of the RVE, including the volume fraction of constituents, geometric characteristics such as orientation and shape of constituents, and morphological characteristics such as the interaction of different constituents in the RVE. The analytical expression (20) can replace the microscale boundary value problem (10), modeling hierarchical materials through sequential upscaling from the lowermost to the macroscale.

#### *Remark 1*

A typical topology optimization problem intends to find the optimal distribution of one material as opposed to voids denoted by a “0-1” integer parametrization (often called black and white design). This problem is ill-posed as non-convergent finer geometric details are obtained with mesh refinement (Allaire and Aubry 1999). The existence of such solutions relies on relaxation, that is, replacing integer variables with density-like continuous variables. The relaxation is achieved by “homogenization / interpolation” between solid material and void. One such example is the famous solid isotropic material with penalization (SIMP) model. Bendsèe and Sigmund showed in Bendsøe and Sigmund (1999) that these artificial interpolation models fall within a framework of micromechanics-based models in many physically realizable circumstances. Thus, the relaxation is naturally built in our continuum micromechanics-based homogenization approach. This allows us to use gradient-based optimization approaches as outlined in this paper.

## Concurrent material and structure optimization in a micromechanics framework

In this section, we formulate a minimum compliance (or maximum stiffness) problem for concurrent material and structure optimization, departing from Xia and Breitkopf (2014), Rodrigues et al. (2002a), and Theocaris and Stavroulakis (1999).

### A minimum compliance formulation based on micromechanical design variables

**Fig. 2:**
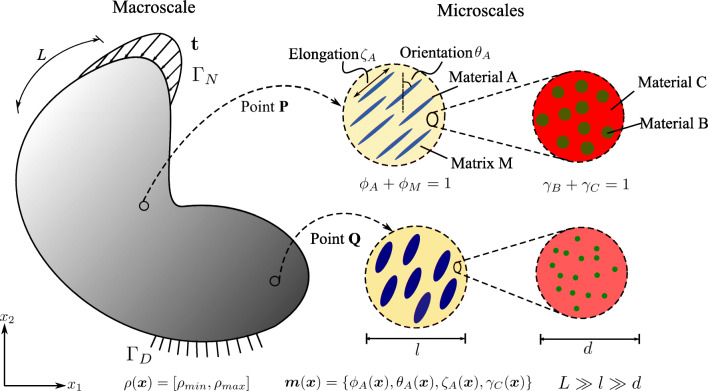
Sketch of a representative problem for material optimization in a continuum micromechanics framework

As illustrated in Fig. 2, we assume a fixed reference domain *Ω* subjected to traction ***t*** at the Neumann boundary *Γ*_*N*_ and prescribed displacements at the Dirichlet boundary *Γ*_*D*_ with a body force field ***b***. At each material point ***x***, microstructural heterogeneities are described by a set ***m***(***x***). The set ***m***(***x***) contains the geometric and mechanical characterization of phases that span multiple well-separated microscales, consisting of volume fraction, material properties, shape, and orientation of the different phases in the hierarchical system. Assuming linear elastic behavior of all constituents, the homogenized macroscale stiffness  at each material point ***x*** depends on the density *ρ*(***x***) and the set ***m***(***x***). Our design vector is therefore [*ρ*(***x***), ***m***(***x***)]^*T*^.

We write a minimum compliance problem in the displacement-based formulation as:
21where *U* denotes the space of kinematically admissible displacement fields $ \bar {\boldsymbol {u}} $, and $ \boldsymbol {E}(\bar {\boldsymbol {u}}) $ denotes the linearized strains.  and *E*_*ad*_ define the set of admissible design variables at the macroscale and microscales, respectively, with possible design constraints. The admissible set  that seeks a limit on the total material mass *M*_*req*_ available for design can be written as:
22where $ \rho _{{\min \limits }} $ and $ \rho _{{\max \limits }} $ are the bounds on the macroscale material density *ρ*.

The definition of the admissible set *E*_*ad*_ is again illustrated via the multiscale configuration shown in Fig. 2. We observe a well-separated three-scale hierarchical system with three base constituent materials denoted as Materials A, B, and C with densities *ρ*_*A*_, *ρ*_*B*_, and *ρ*_*C*_, respectively. At a material point P, the volume fraction of Materials B and C at the lowermost scale are *γ*_*B*_ and *γ*_*C*_ such that *γ*_*B*_ + *γ*_*C*_ = 1. Material B forms spherical inclusions in the matrix of Material C at this scale. The homogenized material from this scale forms the matrix M that hosts Material A inclusions with the orientation *𝜃*_*A*_ and the elongation ratio *ζ*_*A*_ at the mesoscale. The density of the matrix M is simply *ρ*_*M*_ = (*γ*_*B*_*ρ*_*B*_ + *γ*_*C*_*ρ*_*C*_). The volume fractions of Material A and matrix M are *ϕ*_*A*_ and *ϕ*_*M*_ with *ϕ*_*A*_ + *ϕ*_*M*_ = 1. The microstructure characterization field set ***m***(***x***) is {*ϕ*_*A*_(***x***), *𝜃*_*A*_(***x***), *ζ*_*A*_(***x***), *γ*_*C*_(***x***)}.

We can thus write the admissible set *E*_*ad*_ as:

23 $$ \begin{array}{@{}rcl@{}} E_{\textit{ad}} &=& \left\{ \boldsymbol{m}(\boldsymbol{x})  |  \rho(\boldsymbol{x}) = \rho_{A} \phi_{A} (\boldsymbol{x}) + \rho_{M} (\boldsymbol{x}) (1 - \phi_{A} (\boldsymbol{x})), \right.\!\!\!\!\!\\ 0 &<& \phi^{{\min}}_{A} < \phi_{A}(\boldsymbol{x}) < \phi^{{\max}}_{A} \leq 1,  \\ \rho_{M} (\boldsymbol{x}) &=& \rho_{B} (1-\gamma_{C}(\boldsymbol{x})) + \rho_{C}  \gamma_{C}(\boldsymbol{x}), \\ 0 &<& \gamma^{{\min}}_{C} < \gamma_{C}(\boldsymbol{x}) < \gamma^{{\max}}_{C} \leq 1,\\ &&\theta_{A} (\boldsymbol{x}) \in [-\pi/2,\pi/2], \\ &&\left.\zeta_{A} (\boldsymbol{x}) \in [1,\zeta^{{\max}}], \boldsymbol{x} \in {\varOmega} \right\}. \end{array} $$

Here, the volume fraction of Material A is bounded by $ \phi _{A}^{{\min \limits }} $ and $ \phi _{A}^{{\max \limits }} $, and the volume fraction of Material C is bounded by $ \gamma _{C}^{{\min \limits }} $ and $ \gamma _{C}^{{\max \limits }} $ at their respected scales. Also, the elongation ratio of the Material A inclusions is bounded by $ \zeta ^{{\max \limits }} $. These bounds may reflect additive manufacturing constraints on multimaterial composite systems or biological constraints in natural materials. We emphasize again that the multiscale configuration of Fig. 2 is used for the purpose of illustration, but is easily generalized to cover any other multiphase hierarchical system.

### Decomposition into master and slave problems

We note that for a given macroscale density field *ρ*(***x***), the admissible set *E*_*ad*_ is defined pointwise in the domain *Ω*. It allows us to decompose the design formulation (21) as follows:
24

The variational structure of (24) corresponds to a saddle point problem with respect to the admissible set *E*_*ad*_ and the space of kinematically admissible displacements *U*. Lipton worked out in detail and proved the essential conditions that are required for this property to hold (Lipton 1994). This saddle point nature allows us to interchange the second and third operators (*max* and *min*). This interchangeability along with the pointwise definition of *E*_*ad*_ is crucial for decomposing the problem into material and structure optimization sub-problems (Jog et al. 1994).

In the following, we exploit this property to define “master” and “slave” sub-problems. We rewrite formulation (24) as:
25

We reformulate (25) by defining the pointwise maximum strain energy density *Φ* and splitting it into two sub-problems. The outer “master” problem is:
26The pointwise maximum strain energy density sub-problem or “slave” problem is:
27

A combination of (26) and (27) constitutes the concurrent material and structure optimization model. For a given material density distribution *ρ*(***x***), the maximization problem (27) determines the stiffest material microstructure configuration for the evaluated macroscale strain at each material point ***x***. The minimization problem in (26) looks for the kinematically admissible equilibrated displacement field for a given density distribution *ρ*(***x***). The locally optimum strain energies *Φ* in (26) are driven by the pointwise maximization problems in (27) that again depend on the displacement field solution $\bar {\boldsymbol {u}} $. This interdependency makes the equilibrium problem a constitutively nonlinear elasticity problem. Finally, the outer maximization problem (26) seeks the optimal material distribution *ρ*(***x***) in the domain *Ω*.

#### *Remark 2*

The current formulation decomposes the structure and material optimization problem by exploiting the saddle point property of the variational structure of compliance minimization. This decomposition is possible, albeit not straightforward, if other optimality criteria based on, e.g., minimal mass, stress, displacement control, or natural frequency are used. In this context, a general multiscale optimization formulation that decomposes structure and material level problems according to a general optimality criterion was recently presented in Sivapuram et al. (2016). Our approach could be integrated in such a formulation, replacing computationally costly computational homogenization calculations by analytical micromechanics-based estimates

## Finite element discretization and implementation

In this section, we focus on the finite element discretization of the concurrent material and structure optimization formulation and corresponding algorithmic aspects. This includes the treatment of the nonlinearity that results from the interaction between material-scale and structure-scale optimization, and a review of macroscale density optimization, including essential sensitivity analysis. For illustration purposes, we continue to write out our formulation for the special case of the multiscale configuration shown in Fig. 2, but emphasize again that it is easily generalized to cover any other multiphase hierarchical system. In the following, we use vector-matrix notation to represent the introduced quantities, consistent with standard finite element literature (Hughes 2000). However, we keep the same symbols for the respective vector-matrix notation.

### Master problem: structure optimization

We discretize the concurrent material and structure design formulation presented in Section 3 with standard finite elements (Hughes 2000). To this end, we split the domain *Ω* into *N*_*e*_ finite elements, where each element has *N*_*gp*_ Gauss quadrature points. For our example material in Fig. 2, the topology design variables [*ρ*(***x***), ***m***(***x***)]^*T*^ can now be defined elementwise as:

28 $$ \begin{array}{@{}rcl@{}} \boldsymbol{\rho} &= & [\rho_{1}, \rho_{2},\rho_{3}, ..., \rho_{N_{e}}]^{},\\ \boldsymbol{m} &= &  [ ({m^{1}_{1}},..,m^{N_{\textit{gp}}}_{1} ), ({m^{1}_{2}},..,m^{N_{\textit{gp}}}_{2} ), ..., (m^{1}_{N_{e}},..,m^{N_{\textit{gp}}}_{N_{e}} ) ]^{}, \\ {m^{x}_{j}} &= &  [\phi^{x,j}_{A}, \theta^{x,j}_{A} , \zeta^{x,j}_{A} , \gamma^{x,j}_{C} ]^{},   x = 1,...,N_{\textit{gp}}, j = 1,...,N_{e}.\\ \end{array} $$

The macroscale density *ρ*_*j*_ is assumed to be constant in each element, with *j* being the element index. The microscale design variable set ***m*** is defined at each (macroscale) Gauss point, with *x* being the Gauss point index. The microscale design variable $ {m^{x}_{j}} $ consists of volume fraction $\phi ^{x,j}_{A}$, orientation $ \theta ^{x,j}_{A} $, elongation $\zeta ^{x,j}_{A} $, all for Material A , and volume fraction $ \gamma ^{x,j}_{C} $ of Material C.

We can relate the macroscale stress **Σ** with the macroscale strain ***E*** at a Gauss point ***x*** inside element *j* in terms of the design variables *ρ*_*j*_ and $ {m^{x}_{j}} $ as:
29where  is the homogenized stiffness at this point. Interested readers can find the analytical expression for , derived from continuum micromechanics, in Appendix A. The macroscale strain ***E***(***x***) at a point ***x*** inside element *j* is approximated by the element displacement vector $\boldsymbol {\bar {u}}_{j} $ of the element *j* and the strain-displacement matrix ***B***(***x***) that contains shape function information:

30 $$ \boldsymbol{E}(\boldsymbol{x}) \approx \boldsymbol{B}^{}(\boldsymbol{x})  \boldsymbol{\bar{u}}_{j} .  $$

Denoting the compliance of the system with *f*_*c*_(***ρ***), we obtain the following discretized formulation of the “master” problem (26) utilizing the definitions (28) to (30):

31 $$ \begin{array}{ll} \min_{\boldsymbol{\rho}}: &  f_{c}(\boldsymbol{\rho}) = \boldsymbol{f}^{T}_{\textit{ext}}\boldsymbol{\bar{u}}\\ \text{s.t.}: &  \boldsymbol{\bar{r}}(\boldsymbol{\bar{u}}, \boldsymbol{\rho}, \boldsymbol{\bar{m}}) = 0 \\ &\!\!\!\!\!\! M(\boldsymbol{\rho}) = \sum\limits_{j=1}^{N_{e}} \rho_{j} |{\varOmega}_{j}| = M_{\textit{req}} = M_{\textit{frac}} \times \rho_{C} \times|{\varOmega}| \\ &  \rho_{j} \in [\rho_{{\min}},\rho_{{\max}}] ,  \forall j = 1,2,...,N_{e}. \end{array}  $$

The quantities in (31) require further explanation: $ \boldsymbol {f}^{}_{\textit {ext}} $ is the external force vector, $ \boldsymbol {\bar {u}} $ is the global displacement vector that represents the converged macroscale displacement solution, and *M*(***ρ***) is the total mass of the occupying domain, where *ρ*_*j*_ and |*Ω*_*j*_| are the density and the volume of element *j*, respectively. The total available mass *M*_*req*_ can be expressed in terms of fraction *M*_*frac*_ with respect to the mass when the densest material occupies the complete domain. The force residual at the macroscale scale is defined as:
32where *w*_*x*_ contains the Gauss point weight and the determinant of the Jacobian matrix and $\boldsymbol {\bar {u}}_{j} $ is again the element displacement vector of element *j*. We observe that the microstructure design variables ***m*** are implicitly accounted for by $\boldsymbol {\bar {r}}$. At each Gauss point ***x***, the homogenized stiffness  is evaluated based on a microstructure configuration $\boldsymbol {\bar {m}}$ that maximizes the local strain energy.

Identifying the term in the bracket inside (32) as the element stiffness matrix for element *j*, we can rewrite (32):

33 $$ \boldsymbol{\bar{r}}(\boldsymbol{\bar{u}}, \boldsymbol{\rho}, \boldsymbol{\bar{m}}) = \boldsymbol{f}^{T}_{\textit{ext}} - \boldsymbol{K}(\boldsymbol{\rho},\boldsymbol{\bar{m}}^{}(\boldsymbol{\bar{u}})) \boldsymbol{\bar{u}},  $$

where ***K*** denotes the global stiffness matrix of the system. For a given macroscale density distribution ***ρ***, the microstructure ***m*** defined at each Gauss point is optimized with respect to the macroscale strains evaluated at each Gauss point according to (27). The optimized microstructure configuration $ \boldsymbol {\bar {m}}^{} $ updates the macroscale constitutive behavior that is incorporated in ***K***.

### Slave problem: material optimization

For a given material distribution ***ρ*** and displacement solution $ \boldsymbol {\bar {u}} $, the formulation of a “slave” problem (27) at a Gauss point ***x*** inside element *j* is:
34where ***σ*** and *ε* are the stress and strain fields inside the microscale RVE region *Ω*_*y*_ situated at the Gauss point ***x***. It is important to note that we keep (34) in tensor notation, considering its direct relation with Section 2. The optimized configuration $ \bar {m}^{x}_{j} $ that maximizes the strain energy density is sought in the microscale design variable space $ {m}^{x}_{j} = [\phi ^{x,j}_{A}, \theta ^{x,j}_{A} , \zeta ^{x,j}_{A} , \gamma ^{x,j}_{C}]$. All the microstructure constraints directly follow from the admissible set *E*_*ad*_ defined in (23).

The first two conditions in (34) represent equilibrium and strain compatibility in the microscale RVE as discussed in Section 2.2, and correspond to the strong form of the variational statements (10) and (11). If the microstructure is deterministic, these equations can be discretized and solved using the finite element method. The volumetric average of the stress ***σ*** over the microscale RVE volume uses the macroscale stress, and hence the homogenized stiffness . In Section 2.3, we derived the estimates for  based on continuum micromechanics, when only partial statistical information about the microstructure is available (see also Appendix A). The analytical expression (53) renders (34) a straightforward “discretization-free” constraint optimization problem that can be solved by standard gradient-based methods (Boyd et al. 2004). Interested readers are referred to Appendix A for a brief discussion on solving the microscale optimization problem. The solution of (34) at each Gauss point yields the optimized microstructure configuration set $ \boldsymbol {\bar {m}}^{} $.

### Interaction of material and structure scales

**Fig. 3:**
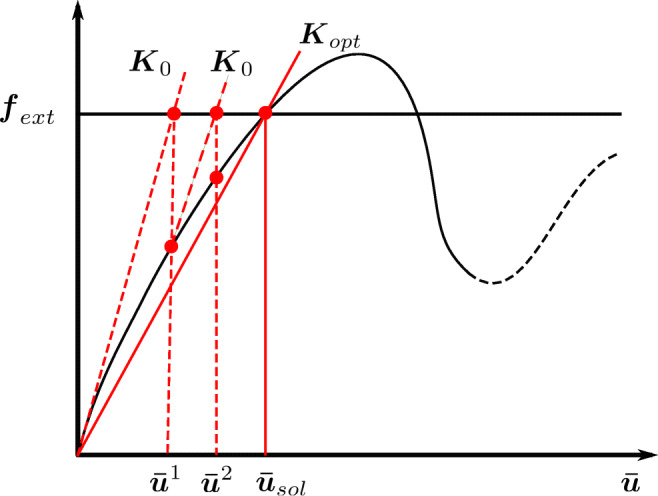
Quasi-Newton method with initial stiffness that resolves the nonlinearity based on the interaction of material and structure scales

Due to the interaction of the material and structure scales, the equilibrium equation (33) is nonlinear. Our approach to resolve this nonlinearity is based on Xia and Breitkopf (2014). For a given macroscale density distribution, we intend to find the equilibrium solution that minimizes the compliance of the system. We may find many possible solutions of the microstructure variable set ***m*** that can potentially satisfy the macroscale equilibrium. We illustrate this point in Fig. 3. For a given external force vector ***f***_*ext*_, many equilibrium solutions exist at the structure scale, depending on different macroscale variable sets. However, we are only interested in the admissible equilibrium solution that minimizes the compliance (or maximizes the stiffness) of the system, lying on a representative load-displacement curve. We can write this preposition mathematically as follows:

35 $$ \min\limits_{\boldsymbol{\bar{u}} \in \boldsymbol{\bar{u}}_{\textit{sol}}}  \boldsymbol{f}_{\textit{ext}}^{T} \boldsymbol{\bar{u}},   \text{s.t.} : \boldsymbol{K}_{\textit{sol}}(\boldsymbol{\rho},\boldsymbol{\bar{m}}(\boldsymbol{\bar{u}}_{\textit{sol}})) \boldsymbol{\bar{u}}_{\textit{sol}} = \boldsymbol{f}_{\textit{ext}} ,  $$

where $ \boldsymbol {\bar {u}}_{\textit {sol}} $ is the set of admissible equilibrium displacement solutions and ***K***_*sol*_ is the stiffness matrix of the system.

It is apparent from Fig. 3 that the solution $\boldsymbol {\bar {u}}$ that satisfies (35) is the first converged displacement solution highlighted by the solid-red line. We can iteratively find this solution, using a quasi-Newton method based on the initial stiffness ***K***_0_. We illustrate this procedure in Fig. 3 by the displacement solutions shown in dashed-red lines. Given the known solution $\boldsymbol {\bar {u}}^{k}$ at the *k*^*th*^ iteration, we find the increment in the solution $ {{\varDelta }}\boldsymbol {\bar {u}}^{k} $ as:

36 $$ \boldsymbol{K}_{0} {{\varDelta}}\boldsymbol{\bar{u}}^{k} = \boldsymbol{f}_{\textit{ext}} - \boldsymbol{f}_{\textit{int}}^{k} .  $$

The internal force vector $\boldsymbol {f}_{\textit {int}}^{k}$ is evaluated with the known displacement solution $ \boldsymbol {\bar {u}}^{k} $ as:
37$\bar {m}^{x,k}_{j}$ is obtained by solving the microstructure optimization problem (34), where kinematic boundary conditions are derived from the displacement solution $ \boldsymbol {\bar {u}}^{k} $. The iterative solution stops when the displacement convergence criteria is met. The optimum solution of the microscale design variables and the corresponding stiffness at the converged displacement solution $ \boldsymbol {\bar {u}}$ are $ \boldsymbol {\bar {m}} (\boldsymbol {\bar {u}}) $ and $ \boldsymbol {K}_{\textit {opt}} (\boldsymbol {\rho },\boldsymbol {\bar {m}} (\boldsymbol {\bar {u}}))$, respectively. The objective function *f*_*c*_(***ρ***) is:

38 $$ f_{c}(\boldsymbol{\rho}) = \boldsymbol{f}^{T}_{\textit{ext}}\boldsymbol{\bar{u}} = \boldsymbol{\bar{u}}^{T} \boldsymbol{K}_{\textit{opt}} (\boldsymbol{\rho},\boldsymbol{\bar{m}} (\boldsymbol{\bar{u}}))\boldsymbol{\bar{u}}.  $$

### Sensitivity analysis and macroscale design update

The macroscale design problem (31) can be solved by well-established optimization algorithms (Bendsøe and Sigmund 2013; Xia and Breitkopf 2017). First, we need to derive the sensitivity of the objective function with respect to the design variables. Using the adjoint method, we write the sensitivity of the objective function *f*_*c*_ with respect to the macroscale design variable ***ρ*** as Bendsøe and Sigmund (2013):

39 $$ \frac{\partial f_{c}}{\partial \boldsymbol{\rho}} = - \boldsymbol{\bar{u}}^{T} \frac{\partial \boldsymbol{K}_{\textit{opt}} (\boldsymbol{\rho},\boldsymbol{\bar{m}} (\boldsymbol{\bar{u}}))}{\partial \boldsymbol{\rho}} \boldsymbol{\bar{u}}. $$

Using (32), we rewrite the sensitivity for each element *j* with respect to its density *ρ*_*j*_ as:
40

The homogenized stiffness  at each Gauss point inside an element *j* is a function of microscale variables $\phi ^{x,j}_{A}, \theta ^{x,j}_{A} , \zeta ^{x,j}_{A} $, and $\gamma ^{x,j}_{C} $ (see Appendix A). Furthermore, $\phi ^{x,j}_{A}$ and $\gamma ^{x,j}_{C} $ relate to *ρ*_*j*_ via (34). Using the chain rule, we find the first derivative of  with respect to *ρ*_*j*_ as:
41where the partial derivatives of  with respect to $ \phi ^{x,j}_{A} $ and $ \gamma ^{x,j}_{C} $ are evaluated at the optimum solution $ \boldsymbol {\bar {m}} $ of the microscale design variables. We evaluate these derivatives using finite difference approximations. Using (34) and standard algebraic manipulation, we arrive at the following expressions:
42

Sensitivity numbers rank the element sensitivities that are used to update the macroscale design variable. The sensitivity numbers for the compliance minimization problem are:

43 $$ \alpha_{j} = - \frac{\partial f_{c}}{\partial {\rho}_{j}}.  $$

To avoid mesh dependency and checkerboard patterns, the sensitivity numbers are first smoothed with a filtering scheme defined as:

44 $$ \alpha_{j} = \frac{{\sum}_{j^{\prime}=1}^{N_{j}} g_{jj^{\prime}} \alpha_{j}}{{\sum}_{j^{\prime}=1}^{N_{j}} g_{jj^{\prime}}},  $$

where *N*_*j*_ is the set of neighboring elements for which center-to-center distance ${{\varDelta }}(j,j^{\prime })$ to element $ j^{\prime } $ is smaller than the filter radius $ r_{{\min \limits }} $. The weight factor $ g_{jj^{\prime }} $ is defined as:

45 $$ g_{jj^{\prime}} = {\max} \{0, r_{{\min}} - {{\varDelta}}(j,j^{\prime}) \}.  $$

To improve convergence, the sensitivity numbers are further averaged with the sensitivity numbers of the previous design iteration as:

46 $$ \alpha_{j}^{i+1} \rightarrow (\alpha_{j}^{i+1} + {\alpha_{j}^{i}})/2.  $$

The ratio of sensitivity numbers and the mass constraints is written as:

47 $$ {B_{j}^{i}} = \left( \frac{{\alpha_{j}^{i}}}{{\varLambda}^{i} |{\varOmega}_{j} | }\right)^{\eta},  $$

where *Λ*^*i*^ is the Lagrange multiplier corresponding to the total material mass constraint in design update *i*, and *η* is a damping parameter. We emphasize that mesh dependency and convergence are two critical issues for any topology optimization algorithm. The heuristic scheme summarized in (44) to (47) has been shown to overcome these challenges for mesh-susceptible problems such as “0-1” type and path-dependent inelastic designs (Xia et al. 2018; Xia et al. 2017).

The macroscale density is updated using the well-known optimality criteria method (Sigmund 2001):

48 $$ \scalebox{0.96}{ $\rho_{j}^{i+1} = \begin{cases} {\max} (\rho_{{\min}}, {\rho_{j}^{i}} - \mu ) & \text{if}  {\rho_{j}^{i}} {B_{j}^{i}} \leq {\max} (\rho_{{\min}}, {\rho_{j}^{i}} - \mu ) \\ {\min} ({\rho_{j}^{i}} + \mu, \rho_{{\max}}) & \text{if}  {\min} ({\rho_{j}^{i}} + \mu, \rho_{{\max}}) \geq {\rho_{j}^{i}} {B_{j}^{i}} \\ {\rho_{j}^{i}} {B_{j}^{i}}       & \text{otherwise} \end{cases}$} $$

To prevent a singular global stiffness matrix, the lower limit $ \rho _{{\min \limits }} $ on *ρ*_*j*_ is limited by a small value of 0.001. The maximum possible element density, $\rho _{{\max \limits }} $, depends on the density of the constituents at the microscales and the prescribed bounds in (23). *μ* is a small move parameter that improves the stability, for instance by preventing multiple holes appearing and disappearing during optimization. The Lagrange multiplier *Λ*^*i*^ is updated using the bisection method to satisfy the mass constraint. The design iterations stop when the density convergence criteria is met.

### Algorithmic framework

We cast our developments in the algorithmic framework summarized in Algorithm 1 that mainly consists of three blocks. The outer block represents the macroscale structure optimization iterations using the optimal-criteria method. It stops when the macroscale density ***ρ*** reaches convergence. The innermost block optimizes the microstructure with respect to the microscale design variables ***m*** for all Gauss points with the prescribed macroscale strain ***E***(***x***). The middle block combines the structure and material scales and solves the boundary value problem for displacements for a given distribution of macroscale density, following our discussion in Section 4.3. 
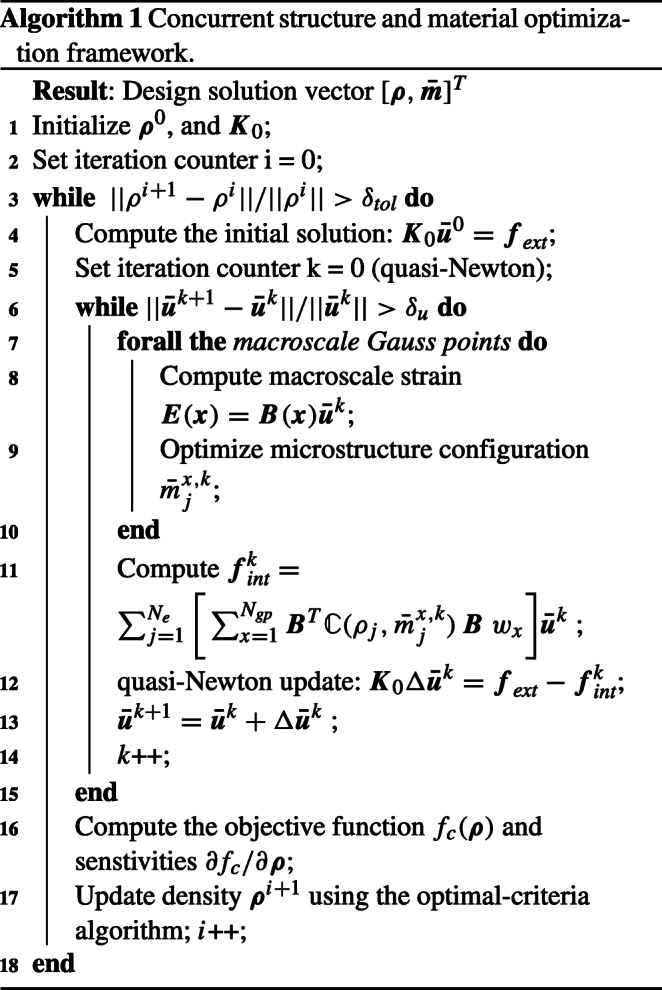


We note that in our context the optimal design can take any value of macroscale density within the allowable range or so-called gray intermediate densities. However, the design framework can be modified for the discrete topology optimization setting with 0-1 type designs. We also note that bi-directional evolutionary structural optimization (BESO) and level-set methods (Huang and Xie 2008; Xia et al. 2018; Sethian and Wiegmann 2000; Allaire et al. 2004) could replace the optimality criteria method in the current framework for 0-1 type design problems.

### Computational cost

Integrating homogenization estimates based on continuum micromechanics in concurrent structure and material optimization leads to an algorithmic framework whose computational cost is independent of the number of hierarchical length scales involved. We briefly illustrate this significant advantage via a qualitative analysis of the underlying computational complexity.

With *n*_*macro*_ macroscale optimization iterations, $ n^{\textit {eq}}_{\textit {itr}} $ average quasi-Newton nonlinear equilibrium iterations, and *n*_*gp*_ gauss points in the macroscale domain, the overall CPU time scales as:
49Here, *T*_*μ*_ is the average CPU time required for the solution of one microscale analysis and optimization problem. We note that $ n_{\textit {macro}},n^{\textit {eq}}_{\textit {itr}}$, and *n*_*gp*_ in (49) are barely modifiable for a required macroscale spatial discretization. This restriction leaves us with *T*_*μ*_ for reducing *T*_*CPU*_.

With nested computational homogenization in the sense of standard FE^2^ type approaches, the computational complexity of *T*_*μ*_ for *s* microscale levels (*s* = 2 in Fig. 2) can be approximately written as:
50where $n^{\mu (s)}_{\textit {gp}} $ and $ n^{\mu (s)}_{\textit {micro}} $ denote the number of quadrature points in the spatial discretization of the RVE at the *s*^*th*^ scale and the number of microstructure optimization iterations required, respectively.

With continuum micromechanics, *T*_*μ*_ is essentially the time to solve the “slave” problem (34). As discussed, this can be achieved by solving a straightforward constraint optimization problem that seeks the solution in the microscale design variable space, using fast gradient-based optimization methods (Boyd et al. 2004). The solution of a slave problem is equivalent to solve a set of (*n* + *p*) nonlinear equations with (*n* + *p*) variables, where *n* and *p* are the total number of design variables and the total number of equality constraints, respectively. The addition of another hierarchical scale potentially increases the number of design variables and constraints in the “slave” problem. However, a few additional design variables do not lead to an exponential increase in computational cost required to solve (34). We detail this computational aspect in Appendix A. We can therefore assume that in our approach, *T*_*μ*_ in (49) scales linearly with each scale characterization.

**Fig. 4:**
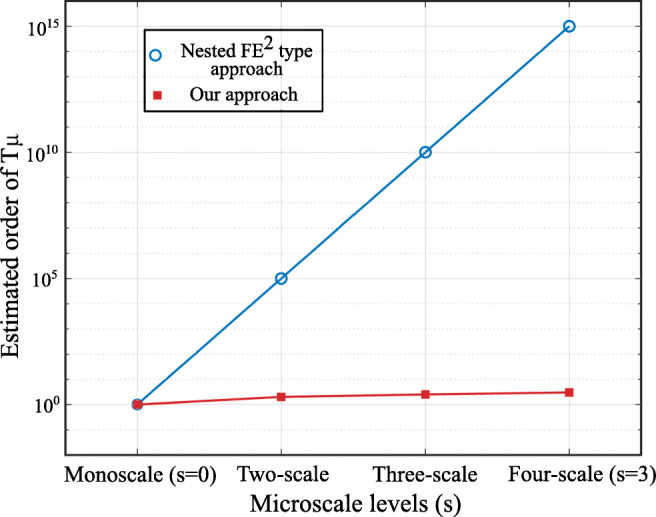
Computational cost of one microscale optimization problem for different numbers of hierarchical scales

Focusing on the solution of one microscale analysis and optimization problem, Fig. 4 compares the scaling of the estimated order of the computational cost with increasing number of materials scales in the two approaches discussed. We observe that for computational homogenization, even a simple two-scale (*s* = 1) problem results in the explosion of the computational expense. For example, given a discretization in each microscale RVE of $ n^{\mu (1)}_{\textit {gp}} \approx 40 \times 40 \times 4 $ and an average number of optimization iterations $n^{\mu (1)}_{\textit {micro}} \approx 20 $, the total computational expense *T*_*μ*_ is of order $ \sim 10^{5} $. If we assume the same RVE discretizations and iteration numbers across multiple scales, we observe in Fig. 4 that the total increases exponentially when *s* > 1. In contrast, the computational cost in our approach remains within the same order of magnitude, even when *s* > 1.

## Numerical examples

In this section, we first define two test examples with hierarchical systems at the material level that are suitable to illustrate the computational efficiency and validity of our concurrent material and structure optimization framework. We then outline the application of our concurrent framework for optimizing natural multiphase hierarchical systems, using the example of bamboo culm.

### Messerschmitt-Bölkow-Blohm (MBB) beam

**Fig. 5:**
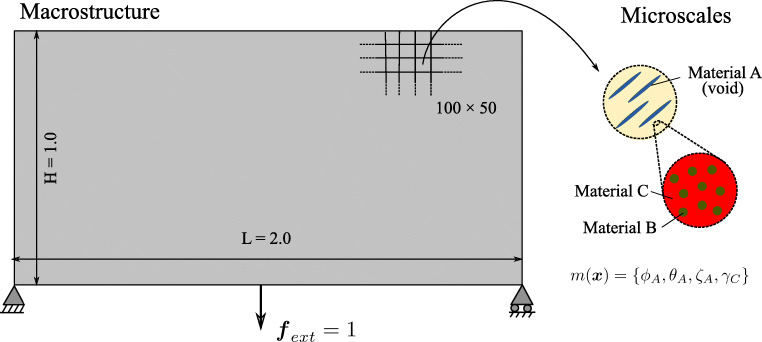
Multiphase hierarchical system I: the MBB beam

We first consider a standard bridge-type structure that is illustrated in Fig. 5. In a structural optimization context, the macroscale configuration is often referred to as Messerschmitt-Bölkow-Blohm (MBB) design problem. The length and height of the macrostructure are 2.0 and 1.0, respectively. The bottom-left end is pinned, and the bottom-right end has a roller support. The structure is loaded with a vertical point load of magnitude one, applied in the middle of the bottom edge of the structure. We discretize the macroscale structure with a 100 × 50 mesh of 4-node quadrilateral elements, resulting in 5000 macroscale design variables. Each element contains four Gauss points, resulting in 100 × 50 × 4 = 20,000 “slave” problems.

In the scope of this work, we extend the MBB test case at the material level. As illustrated in Fig. 5, we consider a hierarchical system that consists of Materials A, B, and C at two different length scales. Their densities are *ρ*_*A*_ = 0, *ρ*_*B*_ = 0.5, and *ρ*_*C*_ = 1.0, respectively; their Young’s moduli are *E*_*A*_ = 0.0, *E*_*B*_ = 0.5, and *E*_*C*_ = 1.0, respectively; and Poisson’s ratio of all constituents is 0.3. For Material A, the elongation ratio of inclusions ranges from *ζ*_*A*_ = 1 to $ \zeta _{A}^{{\max \limits }} = 5 $, and its minimum volume fraction is *γ*_*A*_ = 0.2. For Material C, the volume fraction at the lowermost scale is allowed to assume any value between $ \gamma _{C}^{{\min \limits }} = 0 $ and $ \gamma _{C}^{{\max \limits }} = 1 $. As a consequence, the macroscale density at each point is restricted within the range of $ \rho ^{{\min \limits }}=0 $ to $ \rho ^{{\max \limits }} = 0.8 $. We conclude that at each Gauss point, the material microstructure is parametrized by the volume fraction $\phi ^{x,j}_{A}$, the orientation $ \theta ^{x,j}_{A} $, the elongation $\zeta ^{x,j}_{A} $, and the volume fraction $ \gamma ^{x,j}_{C} $, resulting in 80,000 microscale design variables.

The total amount of material mass available cannot fall below *M*_*frac*_ = 0.4. As an initial condition at the macroscale, we assume the maximum possible density $ \rho ^{{\max \limits }} $ in each element. At the material level, we assume an initial microstructure configuration with *ϕ*_*A*_ = 0.1, *𝜃*_*A*_ = 0.0, *ζ*_*A*_ = 1.0, and *γ*_*C*_ = 1.0 at each Gauss point. In each design update, we reduce the target mass fraction by 0.025 until we reach the specified mass fraction *M*_*frac*_ = 0.4. The move parameter *μ* and the damping parameter *η* are set to 0.05 and 0.5. We choose $ r_{{\min \limits }} = 0.075 $ for the design sensitivity filter (45). Given a macroscale density distribution ***ρ***, the quasi-Newton scheme uses the initial stiffness matrix ***K***_0_ for finding the optimum design variables $ \boldsymbol {\bar {m}} $ (see Section 4.3).

**Fig. 6:**
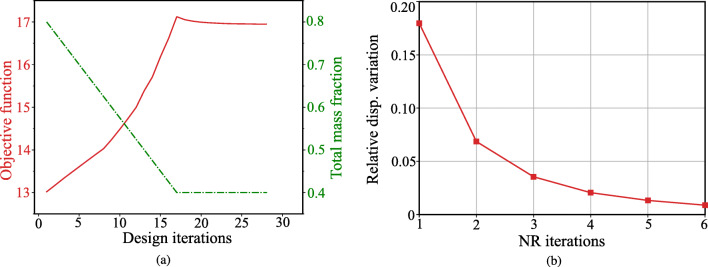
Convergence of macroscale and microscale iterative procedures. **a** Convergence of compliance and mass fraction with respect to number of macroscale design iterations. **b** Convergence of the quasi-Newton method in the 19th design iteration

Figure 6a and b show a convergence plot for the macroscale design updates and the number of quasi-Newton iterations for the macroscale structure problem, respectively. The macroscale design algorithm stops when the relative change in the macroscale density field falls below 0.001. We observe that the algorithm takes 28 density updates to converge to the final design for the MBB problem. The displacement convergence criterion for the quasi-Newton method in each macroscale design iteration is $|| \boldsymbol {\bar {u}}^{k+1} - \boldsymbol {\bar {u}}^{k} ||/ ||\boldsymbol {\bar {u}}^{k}|| < 10^{-2} $. For each macroscale density update iteration, it takes 4 to 8 quasi-Newton iterations to reach the macroscale equilibrium solution. A slave problem takes about 0.003 s on a Mobile Dell Precision 5550 workstation. The total computational time for the macroscale design problem is approximately 2 h with approximately 4 min per design iteration.

**Fig. 7:**
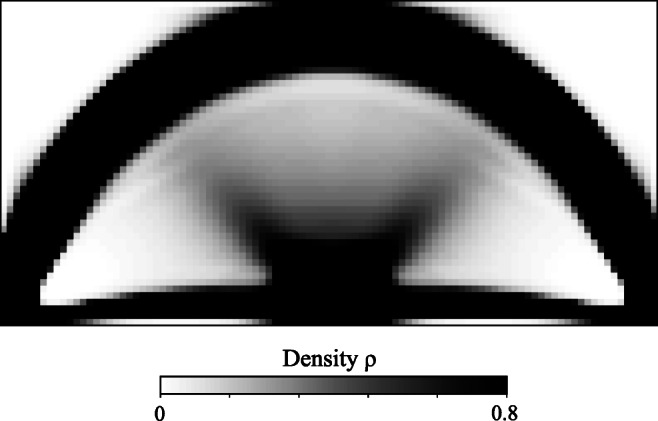
Optimum density distribution for the MBB problem

**Fig. 8:**
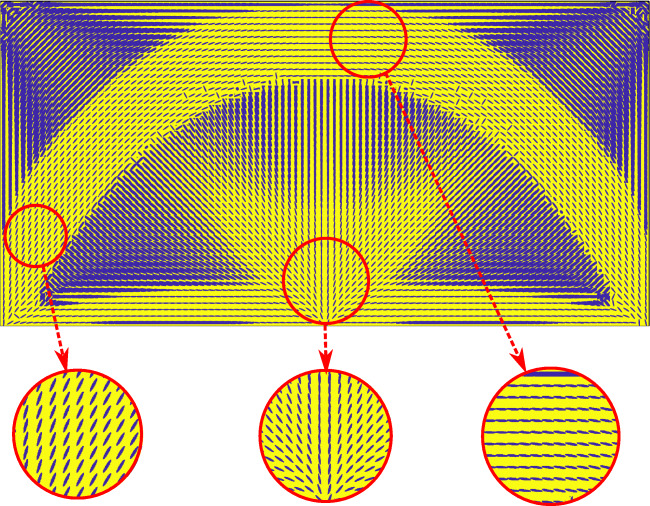
Optimized microstructure at the mesoscale for the MBB problem

**Fig. 9:**
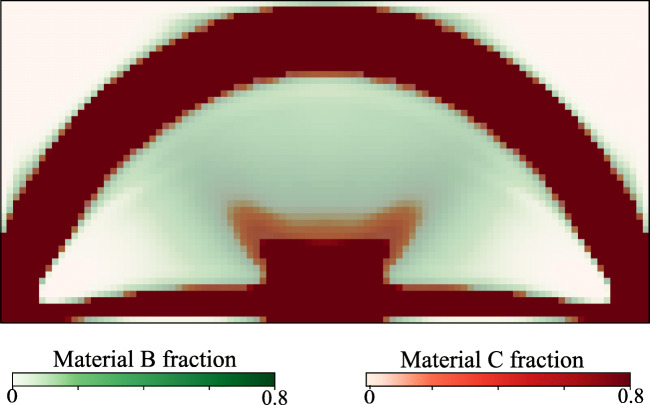
Optimized volume fractions of Material B and Material C for the MBB problem

Figures 7, 8, and 9 illustrate the final design of the MBB problem, including the optimized microstructure configurations. The macroscale density plotted in Fig. 7 shows a large diffuse gray region that maximizes the compliance by optimally distributing the constituents at different scales. The result resembles natural materials such as bones and plants that often exhibit dense cortical-type regions supported by diffuse softer material.

Figure 8 illustrates the details of the optimized morphology at the mesoscale. The yellow color represents the matrix material resulting from homogenization of the lowermost scale. The blue color displays the volume fraction, orientation, and elongation of Material A inclusions. We observe that in the main branches, the inclusions are fully elongated and oriented in the direction of the largest principal stress. In the diffuse regions and joints of the main branches, the morphology is more complex, exhibiting gradual changes in the inclusion characteristics.

The equivalent volume fractions of the three Materials A, B, and C at the macroscale satisfy $ \bar {\phi }_{A} + \bar {\phi }_{B} + \bar {\phi }_{C} = 1 $ and can thus be computed as follows: $ \bar {\phi }_{A} = {\phi }_{A} $, $ \bar {\phi }_{B} = (1-{\phi }_{A}) (1 - \gamma _{C}) $, and $ \bar {\phi }_{C} = (1-{\phi }_{A}) \gamma _{C} $. Figure 9 displays the equivalent material volume fraction of Material B and Material C at the macroscale, where we use 60% opacity for both. We can identify regions dominated by Material B and C as well as a mixing zone. As expected, the stronger Material C is deposited in the main branches, whereas the softer Material B concentrates in the transition zones.

**Fig. 10:**
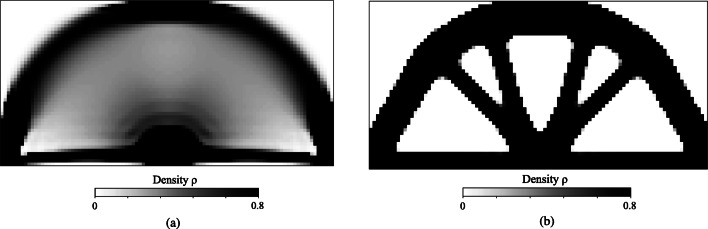
Typical monoscale design using the solid isotropic material with penalization model (SIMP) with different material exponent parameters. **a** Material exponent *p* = 1. **b** Material exponent *p* = 3

For a qualitative comparison, Fig. 10 illustrates typical monoscale designs for the MBB problem that we obtained via the solid isotropic material with penalization model (SIMP) (Bendsøe 1989; Rozvany 2001). In the SIMP method, Young’s modulus is artificially interpolated for intermediate densities as *E* = *ρ*^*p*^*E*_0_, where *p* is the material exponent and *E*_0_ denotes Young’s modulus of the base material. We illustrate two typical optimum density distributions obtained with material exponents *p* = 1 and *p* = 3. The interpolation with *p* = 1 corresponds to the Voigt upper bound material interpolation. The grayscale design with *p* = 1 does not satisfy the Hashin-Shtrikman bound, and, therefore, it cannot be physically realized (Bendsøe and Sigmund 1999). However, the Voigt upper bound interpolation-based designs in topology optimization are popular. Thus, we compare the design shown in Fig. 10a with the corresponding design from our method for insights on the local material adaption. In general, this density layout is similar to the density distribution presented in Fig. 7 with noticeable differences in the diagonal area. In our examples, the material definition is extensive, allowing the redistribution of constituents with local adaption in the morphology. This results in efficient utilization of the lighter Material B and a local morphology based on Material A inclusions as detailed in Figs. 8 and 9. However, in the SIMP design, material configuration choices are limited to a simple density-based parametrization that results in a diffuse distribution of the material in the diagonal area.

### Cantilever beam

**Fig. 11:**
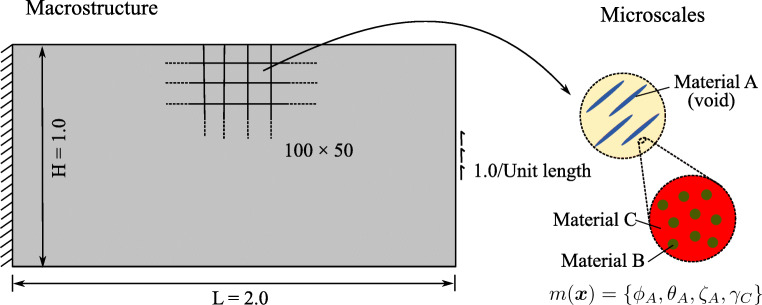
Multiphase hierarchical system II: the cantilever problem

As a second test, we define the cantilever design problem illustrated in Fig. 11. The length and height of the macrostructure are 2.0 and 1.0, respectively. The left edge is fixed, and the central 4% of the right edge are loaded with a traction of magnitude 1.0 per unit length. We employ the same discretization of the macroscale domain as in the previous example. The total amount of mass available is restricted to *M*_*frac*_ = 0.6. The move parameter *μ*, the damping parameter *η*, and the design sensitivity filter radius $ r_{{\min \limits }}$ are 0.05, 0.5, and 0.06, respectively. The rest of the parameters are the same as in the previous example.

**Fig. 12:**
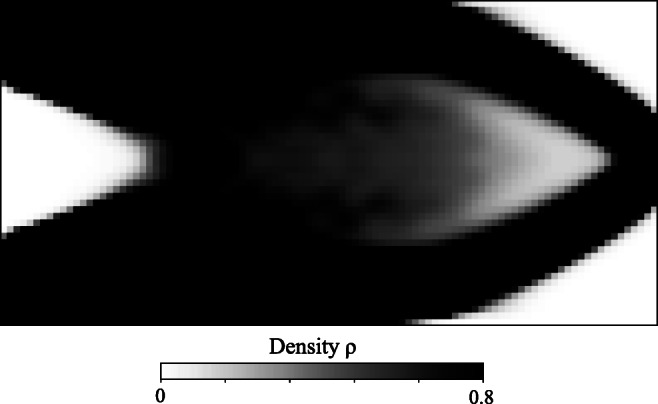
Optimum density distribution for the cantilever problem

**Fig. 13:**
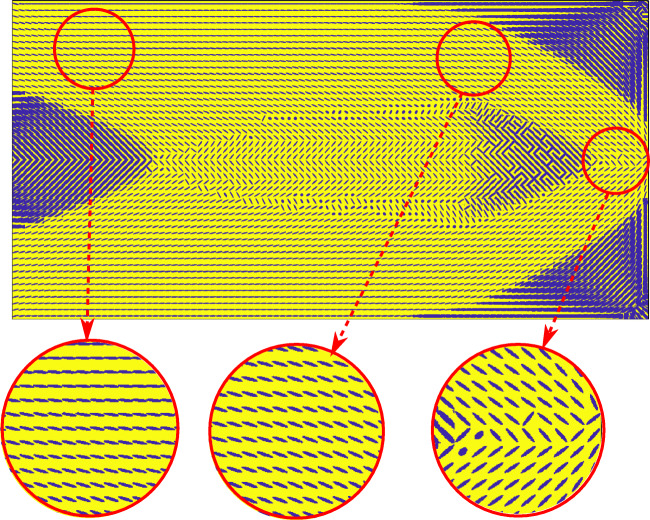
Optimized microstructure at the mesoscale for the cantilever problem

**Fig. 14:**
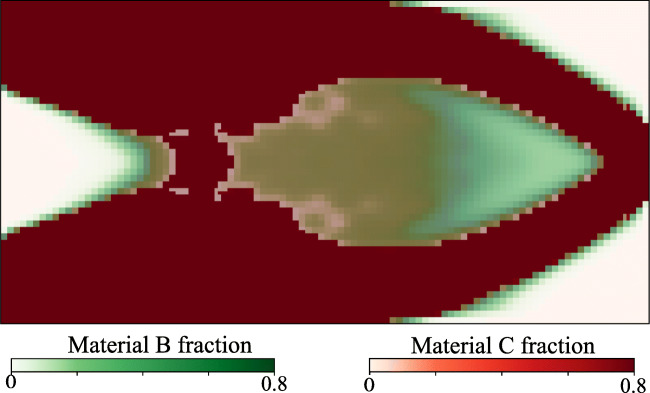
Optimized volume fractions of Material B and Material C for the cantilever problem

We observe in Fig. 12 that the optimized density distribution is qualitatively similar to a standard monoscale variable thickness design. An apparent difference, however, is the significant diffused gray region with complex microstructures, resulting from a complex stress-strain distribution throughout the domain, mainly due to the small length to height ratio. This complex distribution drives the microstructure to adapt itself to achieve optimal performance. Figures 13 and 14 show the morphology of Material A inclusions and the volume fraction distributions of Materials B and C, respectively.

In the diffuse transition regions, the complex strain distributions result in the discontinuity of the flow of the inclusions, as observed in Figs. 13 and 8 in Section 5.1. In these regions, slave problems often have multiple local optima with very close optimal values. For example, an RVE under pure shear has exactly two optimal configurations with inclusion orientation of 45^∘^ and 135^∘^ that have the same optimal value. Therefore, the discontinuity in the flow is a result of locally optimal solutions to slave problems. Adding local connectivity constraints to the slave problems can tackle this issue (Kumar and Suresh 2019; Groen and Sigmund 2018; Allaire et al. 2019).

### Towards hierarchical optimization of bamboo culm

During their growth, the hierarchical composition of biomaterials is subjected to many mechanical, physiological, biological, and phylogenetic constraints. In addition to computationally tractable multiscale analysis, incorporating these constraints represents a main challenge for an optimization algorithm. A few studies have attempted multiscale optimization of biomaterial systems such as bone remodeling and bioinspired functional materials (Rodrigues et al. 2002b; Coelho et al. 2009; Radman et al. 2013). Several obstacles, however, such as high computational cost and phenomenological tuning, have limited many existing approaches in efficiently and accurately modeling self-adaption and growth of biomaterials and other natural hierarchical systems. With the following example, we demonstrate the potential of our optimization framework to overcome these challenges and fill this gap.

**Fig. 15:**
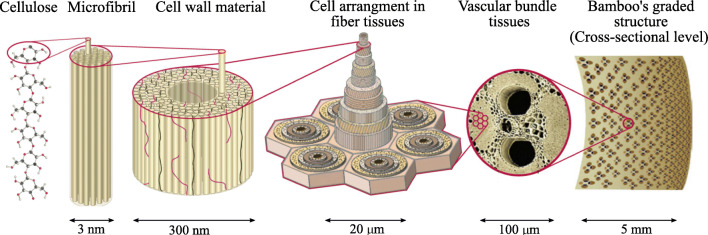
Hierarchical structure of bamboo and its micromechanical representation. Adapted from Wegst et al. (2015) with kind permission from Nature Publishing Group

**Fig. 16:**
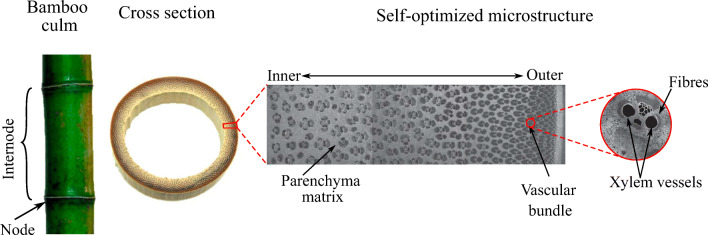
Macroscale anatomy of bamboo with microstructure details through scanning electron microscopy images. The images are reported by Mannan et al. (2017) and reproduced with kind permission from Royal Society Publishing

Bamboo culm materials organize themselves hierarchically across multiple length scales. As illustrated in Fig. 15, these scales range from base constituents such as lignin, cellulose, hemicellulose, and pectin to microstructures at the cell wall, cell, functional-tissue, and cross-section levels (Wegst et al. 2015). Moreover, bamboo does not show secondary growth of tissues and therefore heavily relies on microstructure optimization at the material level (Amada et al. 1996; Liese and Weiner 1996). Figure 16 illustrates that microimaging results confirm the functional optimization in bamboo materials at different length scales (Dixon and Gibson 2014; Mannan et al. 2017). In our previous work, we developed and validated a continuum micromechanics model of bamboo material, building on existing experimental and imaging data about its hierarchical organization (Gangwar and Schillinger 2019). Here, we use this model for accurately assessing material composition and behavior across different scales. For further information on its implementation as relevant to the current paper, the interested reader is referred to Appendix B.

**Fig. 17:**
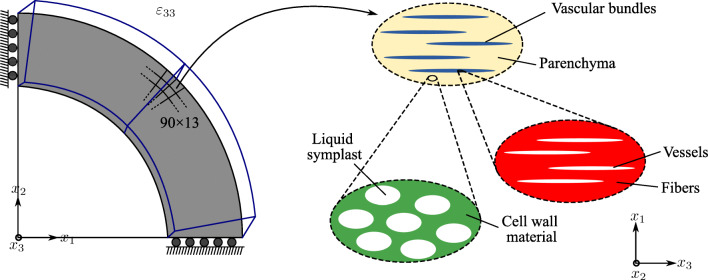
Model problem for the hierarchical optimization of bamboo culm

Figure 17 summarizes the resulting hierarchical optimization problem. We assume that bamboo culm adapts itself to optimally resist bending caused by lateral wind loads. We model one quadrant of the bamboo cross section under symmetry boundary conditions and apply linearly varying radially symmetric axial strains. The outer and inner radius of the quadrant are 90 mm and 72 mm. The quadrant is discretized with a 90 × 13 mesh of 4-node quadrilateral elements, where the aspect ratio of each element is as close to one as possible. This strain distribution is equivalent to the combination of pure bending caused by lateral wind from each direction. With known axial strains and zero out-of-plane shear strains, the problem can be reduced dimensionally such that only in-plane displacements and strains are unknown. For further details on our implementation, the interested reader is referred to Appendix B.

Following our multiscale material model, the microstructure design variables are the cell wall volume fraction *ϕ*_*wall*_ in the parenchyma tissue, the volume fraction *ϕ*_*fib*_ of fibers in the vascular bundles, and the volume fraction *ϕ*_*vb*_ of vascular bundles at the cross-section scale. In bamboo plants, parenchyma tissues and xylem-phloem vessels are responsible for food storage and nutrient-water transport, respectively, and are therefore required to be built in for functional reasons. We incorporate this biological constraint by adopting the bounds on these volume fractions that are experimentally reported in Dixon and Gibson (2014) in the “slave” optimization problem. At the structure scale, the total amount of material is restricted by the reported average density. We interpret this constraint as the limitation posed by the available biological energy required in the synthesis of biomass per unit volume in the bamboo plant.

**Fig. 18:**
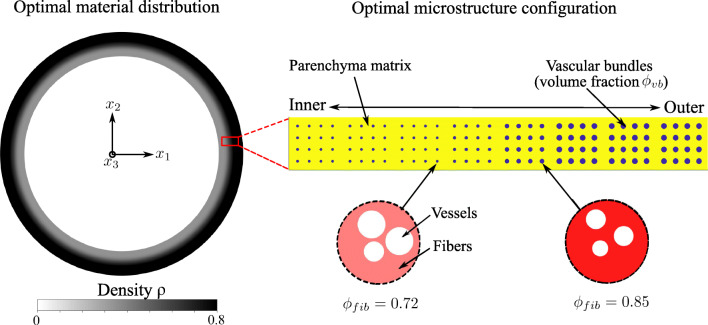
Optimized material distribution and microstructure configuration for the bamboo culm example

Figure 18 illustrates the optimized material distribution at the structure scale and the optimized material microstructure configuration, both obtained with our framework. The optimum density distribution exhibits a strong gradient towards the outer part of the cross section, which is in agreement with the engineering intuition and consistent with experimental observations. Figure 18 also plots the optimized mesoscale morphology along a radial strip of eight 4-node elements. The yellow color represents the parenchyma matrix, and the area of blue circles represents the optimized volume fraction *ϕ*_*vb*_ of vascular bundles at a particular location. We also plot the optimized vascular bundle morphology at two locations, showing different fiber volume fractions *ϕ*_*fib*_. The obtained radial trends for the microscale design variables follow the trends experimentally reported in Dixon and Gibson (2014). We therefore conclude that our framework can quantitatively predict the functional organization and self-adapting mechanism for this natural hierarchical system.

## Summary, conclusions, and outlook

In this article, we presented a concurrent material and structure optimization framework for hierarchical systems that relies on continuum micromechanics estimates for multiscale analysis. The analytical nature of these estimates enables simple constraint optimization problems at the material level that are essentially independent of the number of hierarchical scales, rendering our framework computationally tractable for multiphase hierarchical systems. We successfully verified our optimization framework for two newly defined test problems that are motivated by standard macroscale configurations, but involve hierarchical material definitions at the microscale.

We also applied our framework to simulate self-adapting mechanisms in natural systems. To this end, we integrated an existing continuum micromechanics model for bamboo within our optimization framework. We demonstrated that the resulting optimum design identified by our framework corresponds to material configurations at different scales that are observed in nature. We emphasize that the framework presented in this paper is general and naturally extends to many other engineering applications, involving multiphase hierarchical systems in advanced additive manufacturing and man-made composite material systems.

At this stage of development, our framework is developed for compliance optimization problems with an overall linear elastic material response. Natural systems, however, often exhibit multiscale inelastic behavior and develop dissipation based energy absorption mechanisms against external impacts. This calls for the extension of our framework to inelasticity that originates from the material microscales in hierarchical systems. We think that such an extension can be achieved via continuum micromechanics-based inelastic homogenization methods that are well established for wood, plants, bone, and cement (Fritsch et al. 2009; Pichler and Hellmich 2011; Gangwar and Schillinger 2019; Hofstetter et al. 2008).

## Appendix 1.: Homogenized stiffness through continuum micromechanics

We state the analytical expression for the macroscale homogenized stiffness  at a material point ***x*** in the domain *Ω* (see Fig. 2). The expression at each scale is based on (20). The microstructure characterization variables {*ϕ*_*A*_(***x***), *𝜃*_*A*_(***x***), *ζ*_*A*_(***x***), *γ*_*C*_(***x***)} are defined in Fig. 2. We drop (***x***) from these variables for conciseness of presentation in the following expressions.

At the lowermost scale RVE, Material B forms spherical inclusions in the matrix of Material C. The stiffness tensors for Materials B and C are  and , respectively. The volume fraction of Materials B and C at the lowermost scale are *γ*_*B*_ and *γ*_*C*_ such that *γ*_*B*_ + *γ*_*C*_ = 1. The RVE can be suitably modeled by the Mori-Tanaka scheme. Hence, we assume that  and the Hill tensor , which corresponds to spherical inclusions in the isotropic matrix of Material C. Following (20), we arrive at the homogenized stiffness tensor  of the RVE:

51

This homogenized material matrix M hosts the inclusions of Material A with stiffness . The orientation and elongation ratio of these inclusions are *𝜃*_*A*_ and *ζ*_*A*_, respectively. We estimate the homogenized stiffness of this RVE with the Mori-Tanaka scheme. We assume that  and the Hill tensor , which corresponds to spheroidal inclusions with the elongation ratio *ζ*_*A*_ in the isotropic matrix of material M. We write the macroscale homogenized stiffness  in the co-ordinate system aligned with the inclusion elongation direction following (20). The expression is:

52

The macroscale stiffness tensor  in the global co-ordinate system is obtained with the help of the standard tensor transformation matrix **T** as:

53

The expressions for the Hill tensors  and  are given in Masson (2008). We note that the evaluation of (51) to (53) requires only a few matrix operations involving 3 × 3 matrices, and can thus be considered computationally inexpensive.

Now, we can rewrite the optimization statement (34):

54

where we omit the constraints posed in (34) for clarity of notation. Equation (54) can be simplified further by eliminating *𝜃*_*A*_ (Pedersen 1989; Jog et al. 1994). We know that for a general orthotropic material, the maximum strain energy is obtained by aligning the material axis with the principle strain axes. Therefore, the macroscale strain at each Gauss point entails the optimal material orientation $ \bar {\theta }_{A} $ at the particular Gauss point. This further helps us simplify problem (54) to:

55

This reduced problem can be solved by standard gradient-based fast optimization algorithms. Generally speaking, the solution of (55) is equivalent to finding a solution of the KKT equations that correspond to this constraint problem (Boyd et al. 2004). The KKT equations are a set of (*n* + *p*) nonlinear equations with (*n* + *p*) variables, where *n* and *p* are the total number of design variables and the total number of equality constraints. In this example, *n* = 3 and *p* = 2. It is straightforward to see that each additional scale will result in the addition of a few design variables and constraints. This does not lead to an exponential increase in computational efforts to solve a slave problem. Newton’s method and its variants are computationally efficient to solve these equations. Readers interested in a detailed mathematical analysis of such methods are referred to Boyd et al. (2004).

We use the quasi-Newton method of Broyden, Fletcher, Goldfarb, and Shanno (BFGS) with default parameters implemented in the SciPy library to solve this problem. The total time for one slave problem on a Mobile Dell Precision 5550 workstation is approx. 0.001 s. In addition, all slave problems are independent of each other, and hence are completely parallelizable on a GPU or multi-threaded CPU architecture.

## Appendix 2.: Details on the bamboo application case

We briefly summarize the key components for implementing the bamboo optimization problem in Section 5.3. We refer to Section 4 in Gangwar and Schillinger (2019) for further details on the multiscale characterization and homogenization of the elastic properties of bamboo culm. In the current model, we assume that the cell wall fraction *ϕ*_*wall*_ in the parenchyma tissues, the fiber fraction *ϕ*_*f**i**b*_ in the vascular bundles, and the vascular bundle fraction *ϕ*_*v**b*_ at the cross-section scale are the microstructure design variables at each Gauss point in the domain. Bamboo is a transversely isotropic material, being isotropic in the cross-sectional plane. The macroscale homogenized stiffness tensor as a function of the microscale design variables can be written as:

56

Here,  and  are the homogenized stiffness tensors of parenchyma tissues and vascular bundle tissues, respectively. For the analytical expression of these estimates and other notation, we refer to Gangwar and Schillinger (2019).

We write the discretized optimization statement of the “slave” sub-problems for the bamboo example following (34):

57

where *ρ* is the given macroscale dry density for the relevant finite element. *ρ*_*wall*_ and *ρ*_*fib*_ are the density of cell wall materials and sclerenchyma fibers. The first statement in the constraints simply connects *ρ* with the microscale design variables through the rule of mixture. We adopt the minimum and maximum values of the design variables reported in Dixon and Gibson (2014) as the bounds in the above optimization problem. These bounds for *ϕ*_*wall*_, *ϕ*_*fib*_, and *ϕ*_*v**b*_ are [0.18, 0.22], [0.70, 0.95], and [0.15, 0.60], respectively. This optimization problem is a constraint optimization problem with nonlinear equality constraint. We utilize the sequential least squares programming (SLSQP) method implemented in the SciPy library to solve this problem.

The “master” problem to obtain the optimal material density distribution is rewritten following (31) as:

58

At the macroscale, the total material is restricted by the reported average density *ρ*_*avg*_. In the bamboo problem, we consider a three-dimensional state of stress and strain with zero out-of-plane shear strain components and known axial strains that is *ε*_13_ = *ε*_23_ = 0, and *ε*_33_(***x***) = *f*(*x*_1_, *x*_2_). Essentially, the problem reduces to a two-dimensional case where only in-plane displacements and strains are unknown. Therefore, the problem can be discretized with standard 4-node quadrilateral elements. To enforce known *ε*_33_(***x***) = *f*(*x*_1_, *x*_2_), we modify the strain-displacement matrix as:

$$ \boldsymbol{B} = \begin{bmatrix} \frac{\partial N_{1}}{\partial x} & 0 & 0 & .... \\ 0 & \frac{\partial N_{1}}{\partial y} & 0 & .... \\ 0 & 0 & \frac{\varepsilon_{33}(\boldsymbol{x})}{4} & .... \\ \frac{\partial N_{1}}{\partial y} & \frac{\partial N_{1}}{\partial x} & 0 & .... \\ \end{bmatrix}, $$

and set the axial displacement component in the displacement vector $ \boldsymbol {\bar {u}} $ to one.

The sensitivity analysis for the macroscale design update follows from Section 4.4. The first derivative of  with respect to density *ρ* is replaced with

59

The derivative of  with respect to microscale design variables at the material level is evaluated using finite difference approximations. The move parameter *μ*, the damping parameter *η*, and the design sensitivity filter radius $ r_{{\min \limits }}$ are 0.02, 0.5, and 0.01.
